# 514 The Outcomes of Tracheostomy on Burn Inhalation injuries

**DOI:** 10.1093/jbcr/irac012.145

**Published:** 2022-03-23

**Authors:** Samuel G Ruiz, Salomon Puyana, Shaikh A Hai, Mark G Mckenney, Haaris Mir

**Affiliations:** Kendall Regional Medical Center, Miami Springs, Florida; Tulane University, New Orleans, Louisiana; Kendall Regional Medical Center, Miami, Florida; Kendall Regional Medical Center, Miami, Florida; Kendall Regional Medical Center, Miami, Florida

## Abstract

**Introduction:**

Tracheostomy has been proposed for patients with expected prolonged intubation and it has been shown to be beneficial for trauma patients with severe brain injury; however, the benefit of performing tracheostomy on burn inhalation injuries has not been extensively investigated. Our study aims to determine the outcomes of performing tracheostomy on patients with burn inhalation injuries requiring mechanical ventilation.

**Methods:**

Retrospective review of our institutional burn registry from 2011 to 2019. We compared the outcomes of all burn patients that met our inclusion criteria which included: adequate data recording of inhalation injury within the registry, ventilator support for at least 24 hours, and a TBSA burn injury of < 15%. We stratified the patients into two groups: tracheostomy (group 1) versus no tracheostomy (group 2). Outcome measures included: in-hospital mortality rate, hospital length of stay, ICU length of stay, ventilator days, and ventilator associated pneumonia (VAP). Chi-squared and t-test analyses were used with significance defined as p< 0.05.

**Results:**

A total of 33 burn patients met our inclusion criteria. Group 1 consisted of 10 patients and group 2 consisted of 23 patients. There was no statistically significant difference between the two groups in terms of %TBSA (p =0.24, t-test). There was a significantly higher ICU length of stay at 23.8 days in group 1 compared to 3.16 days in group 2 (p=0.0001, χ2). There was a significantly higher hospital length of stay at 28.4 days in group 1 compared to 5.26 days in group 2 (p=0.0001, χ2). Ventilator days was also significantly higher in group 1 with 20.8 days compared to 2.5 days in group 2. There was no statistically significant difference between the two groups in terms of mortality, however, the incidence of VAP was significantly higher in group 1 than in group 2, with six cases compared to zero cases, respectively(p=0.0001, χ2).

**Conclusions:**

The ideal timing and implementation of tracheostomy with inhalational injury has yet to be determined. In our study, tracheostomy was associated with much longer lengths of stay and pneumonia. The impact of the underlying lung injury, versus the tracheostomy itself on these observations, is unclear. The challenge of characterizing the severity of an inhalation injury based on early visual inspection remains.

